# Human Wharton’s jelly-derived mesenchymal stromal cells promote bone formation in immunodeficient mice when administered into a bone microenvironment

**DOI:** 10.1186/s12967-023-04672-9

**Published:** 2023-11-10

**Authors:** Raquel Cabrera-Pérez, Alexis Ràfols-Mitjans, Ángela Roig-Molina, Silvia Beltramone, Joaquim Vives, Laura Batlle-Morera

**Affiliations:** 1 Servei de Teràpia Cel·lular i Avançada, Blood and Tissue Bank (BST), 08005 Barcelona, Catalonia Spain; 2 Musculoskeletal Tissue Engineering Group, Vall d’Hebron Research Institute (VHIR) and Universitat Autònoma de Barcelona (UAB), 08035 Barcelona, Catalonia Spain; 3 Centre for Genomic Regulation (CRG), Genomic Regulation, Stem Cells and Cancer Program, The Barcelona Institute of Science and Technology, 08003 Barcelona, Catalonia Spain; 4 Medicine Department, Universitat Autònoma de Barcelona (UAB), 08193 Barcelona, Catalonia Spain

**Keywords:** Advanced therapy medicinal product, Bone microenvironment, Bone regeneration, Regenerative medicine, Multipotent mesenchymal stromal cells, Wharton’s jelly

## Abstract

**Background:**

Wharton’s Jelly (WJ) Mesenchymal Stromal Cells (MSC) have emerged as an attractive allogeneic therapy for a number of indications, except for bone-related conditions requiring new tissue formation. This may be explained by the apparent recalcitrance of MSC,WJ to differentiate into the osteogenic lineage in vitro, as opposed to permissive bone marrow (BM)-derived MSCs (MSC,BM) that readily commit to bone cells. Consequently, the actual osteogenic in vivo capacity of MSC,WJ is under discussion.

**Methods:**

We investigated how physiological bone environments affect the osteogenic commitment of recalcitrant MSCs in vitro and in vivo. To this end, MSC of BM and WJ origin were co-cultured and induced for synchronous osteogenic differentiation in vitro using transwells. For in vivo experiments, immunodeficient mice were injected intratibially with a single dose of human MSC and bone formation was evaluated after six weeks.

**Results:**

Co-culture of MSC,BM and MSC,WJ resulted in efficient osteogenesis in both cell types after three weeks. However, MSC,WJ failed to commit to bone cells in the absence of MSC,BM’s osteogenic stimuli. In vivo studies showed successful bone formation within the medullar cavity of tibias in 62.5% of mice treated with MSC, WJ. By contrast, new formed trabeculae were only observed in 25% of MSC,BM-treated mice. Immunohistochemical staining of human COXIV revealed the persistence of the infused cells at the site of injection. Additionally, cells of human origin were also identified in the brain, heart, spleen, kidney and gonads in some animals treated with engineered MSC,WJ (eMSC,WJ). Importantly, no macroscopic histopathological alterations, ectopic bone formation or any other adverse events were detected in MSC-treated mice.

**Conclusions:**

Our findings demonstrate that in physiological bone microenvironment, osteogenic commitment of MSC,WJ is comparable to that of MSC,BM, and support the use of off-the-shelf allogeneic MSC,WJ products in bone repair and bone regeneration applications.

**Supplementary Information:**

The online version contains supplementary material available at 10.1186/s12967-023-04672-9.

## Background

The treatment of bone-related diseases has become a priority in the field of cell therapy and tissue engineering because of their high and increasing prevalence worldwide, particularly in aging societies [1–3]. In this context, the use of multipotent Mesenchymal Stromal Cells (MSC) has emerged as a promising approach to promote bone regeneration in both preclinical and clinical settings [4–12]. However, to ensure the success of these approaches, versatile, off-the-shelf osteogenic tissue engineering products must be developed that follow regulatory pathways specific for advanced therapies [13].

MSCs constitute a heterogeneous population of non-haematopoietic multipotent cells that can be isolated from a number of human body sources and have the capacity to generate bone tissue (in addition to cartilage and fat) both by cell determination and paracrine signalling [4, 14, 15]. Among them, Bone Marrow (BM) is clinically the most widely used tissue source of MSCs in the orthopaedic field because of the permissive osteogenic differentiation potential of MSC,BM [16]. Nevertheless, MSC,BM-based therapies present notable shortcomings that restrict their use in allogeneic treatments such as i) the isolation procedure, which is invasive, painful, and constitutes and important source of morbidity in the donor site; ii) the variability between the age and medical conditions of the donors, which can compromise the regeneration potential of the cells; and iii) the immunogenicity, because although MSC are considered to be negative for HLA-DR, varying percentages of expression are commonly found in MSC,BM cultures [17–20]. In this context, Wharton’s jelly (WJ) MSC (MSC,WJ) are an attractive alternative to MSC,BM due to (i) their accessibility, which allow for their isolation from fresh or well-characterized and cryopreserved umbilical cord tissue [21], which has long been considered as medical waste and thus is associated with minor ethical concerns; (ii) their ease of expansion ex vivo following well-defined banking strategies that result in the generation of off-the-shelf MSC,WJ haplolines that can be specifically selected to maximize immunological compatibility between donor and patient [22]; (iii) their safety profile, which is enhanced due to their newborn and primitive condition [23]; and (iv) their multipotentiality and biological activity to modulate the immune system and tissue-specific resident stem cells, which would permit the generation of a transient regenerated tissue that would serve as a scaffold to potentiate an endogenous remodelling process in advanced stages [24–26].

Although the osteogenic potential of MSC,WJ has been extensively investigated in vitro [22, 27–29]*,* they are recalcitrant to make bone tissue in a timely and efficient manner, especially when compared to permissive MSCs from other tissue sources such as BM or adipose tissue (adipose stem cells, ASC). In this context, several studies based on a direct comparison of the osteogenic potential of MSCs from different origins alone or in combination with different scaffolds have been reported, demonstrating that MSC,WJ exhibited milder expression of osteogenic markers and, thus, a compromised osteogenic commitment [30–35]. Consequently, several different strategies have been established to promote osteogenesis in vitro and in vivo, based on the use of chemical or physical inducers, genetic modification, or in vitro preconditioning methods [29, 32, 36–39]. Despite this, use of MSC,WJ for treatment of bone diseases is still restraint and limited information is available from preclinical and clinical studies demonstrating the adequacy of the osteogenic potential of MSC,WJ as a therapeutic tool in orthopaedics.

We previously reported that, although the intrinsic molecular signature of MSC,WJ apparently counteracts their osteogenic differentiation potential by promoting proliferation instead, secreted factors present in the conditioned media from differentiating MSC,BM cultures strongly enhance MSC,WJ osteogenesis [40]. This observation confirms the readiness of MSC,WJ to generate bone tissue and prompted us to investigate the impact of in vitro bony environments responsible for osteogenic induction of MSC,WJ and in vivo validation.

In the present study, we aimed to demonstrate that MSC,WJ bone differentiation is delayed in vitro due to poor replication of the in vivo osteogenic milieu and, therefore, an intra-bony environment could be enough to guarantee MSC,WJ-promoted osteogenesis.

## Methods

### Cell culture

MSC,BM and MSC,WJ of human origin were expanded in *“expansion media”* composed of Dulbecco’s Modified Eagle’s Medium (DMEM) (Gibco Cat# 31885–023) containing 2 mM glutamine and supplemented with 10% human serum B (hSerB). All cell cultures were maintained at 37 °C and 5% CO_2_ in humidified incubators and media were changed every three-four days. Cell number and viability were determined by the haemocytometer-based trypan blue dye exclusion assay.

### Osteogenic differentiation in cell culture inserts

To determine if the secretome of non-primed MSC,BM could act as a osteogenic stimuli and exert a positive effect on the osteogenic commitment of MSC,WJ, we performed a transwell experiment in which undifferentiated MSC,BM and MSC,WJ were co-cultured using culture inserts, which consisted of tissue culture membranes that allow intercellular communication by diffusion of secreted molecules independent of cell-to-cell contact. Concretely, MSC,WJ and MSC,BM (passage four) were co-cultured using 0.4 μm cell culture inserts (Falcon Cat# 353095). To avoid contamination between cell cultures of different origins, MSC,WJ and MSC,BM were seeded in the appropriate support (well or insert) at 2·10^4^ and 10^4^ cells/cm^2^ respectively, and cultured overnight in different plates to allow cell attachment. To avoid differences due to the cell surface (well versus insert) or the flow directionally, all possible combinations were tested. Next, cell culture inserts were placed into the cultured wells and synchronically induced for osteogenic differentiation in vitro using *“differentiation media”* composed of the StemPro osteogenesis differentiation kit (Gibco Cat# A1007201) supplemented with 100 units/mL of penicillin and 100 µg/mL streptomycin (Penicillin–Streptomycin; Sigma-Aldrich Cat# P4458). Throughout the osteogenic differentiation, cell cultures were maintained for three weeks at 37ºC and 5% CO_2_ in humidified incubators and media were changed every three-four days. Finally, Alizarin Red (AR) (Merck Millipore Cat# 2003999) staining was carried out to identify calcium depositions and assess cell differentiation.

### Genetic engineering of MSC,WJ and bioluminescence assays

For the generation of the MSC,WJ Firefly Luciferase (FFly; FFly-MSC,WJ) cell line, 10^6^ MSC,WJ were electroporated with 5 µg of PX458 plasmid containing AVVS1 sgRNA (Addgene plasmid Cat# 113194, RRID: Addgene_113194) and 5 µg of HDR plasmid P_EI-1a_Firefly luciferase (PpyRE9)_PGK_Puro, generated by the Protein Technologies Unit at CRG. Similarly, for the generation of the MSC,WJ NanoLuc (NLuc; NLuc-MSC,WJ) cell line, 10^6^ MSC,WJ were electroporated with 3 µg of pB-EF1a-NLuc-IRES-Puro (Addgene Cat# 130936, RRID: Addgene_130936) and 9 µg of pBase plasmid, obtained from Piggybac transposase resources Wellcome Sanger Institute. Electroporated pools were selected with 0.1 µg/mL of puromycin, expanded in previously defined *“expansion media”* and cryopreserved prior to assays. For bioluminescence assays, MSC,WJ and eMSC,WJ (containing FFly or NLuc) were plated onto 24-well plates and luciferase substrates D-luciferine (150 µg/mL) and Fluorofurimazine (FFz) (10 µM) were added to the culture media to determine FFly and NLuc activities, respectively. Bioluminescence activity was evaluated ten minutes after addition of the corresponding luciferase substrate, using the Berthold LB 960 luminometer.

### Animal procedures

Immunodeficient NSG™ (NOD.Cg-Prkdcscid Il2rgtm1Wjl/SzJ, Jackson Laboratory, RRID: IMSR_JAX:005557) mice were housed together in Specific Pathogen Free (SPF) conditions, fed a standard diet and allowed access to water ad libitum at the animal facility of the Barcelona Biomedical Research Park (PRBB), accredited by AAALAC International. For intratibial injections, eight to twelve week-old NSG male mice were anaesthetized by intra-peritoneal administration of ketamine/medetomidine and then injected into the right tibia with 5 × 10^5^ MSC-BM (N = 4), MSC,WJ (N = 8), or eMSC,WJ (N = 8) cells resuspended in 10 μl of PBS using a 29G x ½” needle. Non-treated animals (NT, N = 3) and animals injected with PBS (N = 8) were also included as a control (see Additional File 1). The objective of the in vivo study was not to perform a direct comparison of the effect of using MSC,WJ versus MSC,BM but to elucidate the bone formation capacity of non-primed MSC,WJ, which has not been clearly described in the literature to date. Because of this reason as well as to reduce the number of animals in accordance to the 3R principles, we included a smaller number of animals in MSC,BM and non-treated groups. Indeed, sample size of each experimental group and dose of MSC were established according to literature within range published by other authors. Treatment or control groups were assigned randomly. Anaesthesia reversal was performed by subcutaneous injection of Atipamezol. Additionally, Buprenorfine was also subcutaneously injected for analgesic purposes.

For in vivo bioluminescence monitoring, mice were anaesthetized with 1.5% isoflurane for induction and administered via intraperitoneal injection with the respective luciferase substrate: either 50 μL of 30 mg/mL (w/v) Luciferin (FFly) or 50 μL of 8.7 M fluorofurimazine (FFz) (NLuc). At ten min post-substrate administration, dorsal bioluminescence images were taken under 1.5% isoflurane conditions and using the IVIS Spectrum (PerkinElmer) with the following acquisition parameters: open for total bioluminescence, exposure time = 1 min, binning = medium: 4.

Six weeks after treatment, the animals were culled by cervical dislocation and a full necropsy was performed to detect any possible histopathological alterations. Experimental endpoint was stablished according to published data on bone healing/bone regeneration in mice which is stated to occur within 4 weeks. Next, tissue samples including the brain, heart, lung, kidney, liver, spleen, and gonads were collected, immediately frozen in liquid nitrogen, and stored at − 80 °C until performance of biodistribution assays. Treated tibias were immersed in Decalcifier I solution (Leica Cat# 3800400) for histological purposes.

### MSC biodistribution

At the endpoint, MSC biodistribution was assessed by quantitative real-time PCR (qRT-PCR) of human-specific Alu sequences using an ABI PRISM 7900HT detector (Applied Biosystems). To that end, genomic DNA was purified from frozen tissue samples using the QIAamp DNA Mini kit (Qiagen Cat# 51304) according to the manufacturer’s instructions and quantified by spectrophotometry using NanoDrop Lite (Thermo Scientific). Alu sequences were determined using a total amount of 100 ng of genomic DNA and custom-designed hydrolysis probe (56-FAM-CGCCCGGCT-ZEN-AATTTTTGTAT-3IABKFQ) and primers (GGTGAAACCCCGTCTCTACT (forward) GGTTCAAGCGATTGTCCTGC (reverse)) as described elsewhere [41].

### Histological and immunohistochemical staining.

Haematoxylin–eosin staining as well as immunohistochemical detection of human cells (human COXIV) and bone markers (ALP, OCN) were performed in 5-µm thick sections. For immunohistochemistry, sections were rehydrated in a series of xylene and ethanol baths, antigen retrieval was performed with 10 mM sodium citrate solution and endogenous peroxidase activity was inhibited by treatment with 3% hydrogen peroxide. After the washing and permeabilization steps, blocking of non-specific antigens was performed with 2.5% BSA solution. Samples were then incubated with the following primary and secondary antibodies: rabbit anti-human COXIV 1:200 (Cell Signaling Technology Cat# 4850, RRID: AB_2085424), rabbit anti-Osteocalcin 1:200 (Millipore Cat# AB10911, RRID: AB_1587337), rabbit anti-Alkaline Phosphatase 1:200 (Thermo Fisher Scientific Cat# PA5-21332, RRID: AB_11153191), and goat anti-rabbit IgG, HRP 1:500 (Thermo Fisher Scientific Cat# 31460, RRID: AB_228341). Localization of HRP-conjugated antibodies was revealed using eBioscience™ DAB Advanced Chromogenic Kit (ThermoFisher Scientific Cat# 8801–4965-72). Finally, slides were counterstained with Mayer’s Haematoxylin (Sigma-Aldrich Cat# 51275), dehydrated in a series of ethanol and xylene baths, and mounted using DPX mountant for histology (Sigma-Aldrich Cat# 06522).

## Results

### MSC,WJ osteogenic differentiation is accelerated in the presence of MSC,BM

A substantial part of the MSC,BM population that can be found in the stroma of the BM remains in an undifferentiated and multipotent state. To determine whether the osteogenic stimuli produced by non-primed MSC,BM could also potentiate osteogenic differentiation of MSC,WJ, we performed a transwell experiment in which undifferentiated MSC,BM and MSC,WJ were co-cultured using culture inserts. This consisted of tissue culture membranes that allow intercellular communication by diffusion of secreted molecules independent of cell-to-cell contact. The cultures were then synchronically induced for osteogenic differentiation in vitro.

**Fig. 1:**
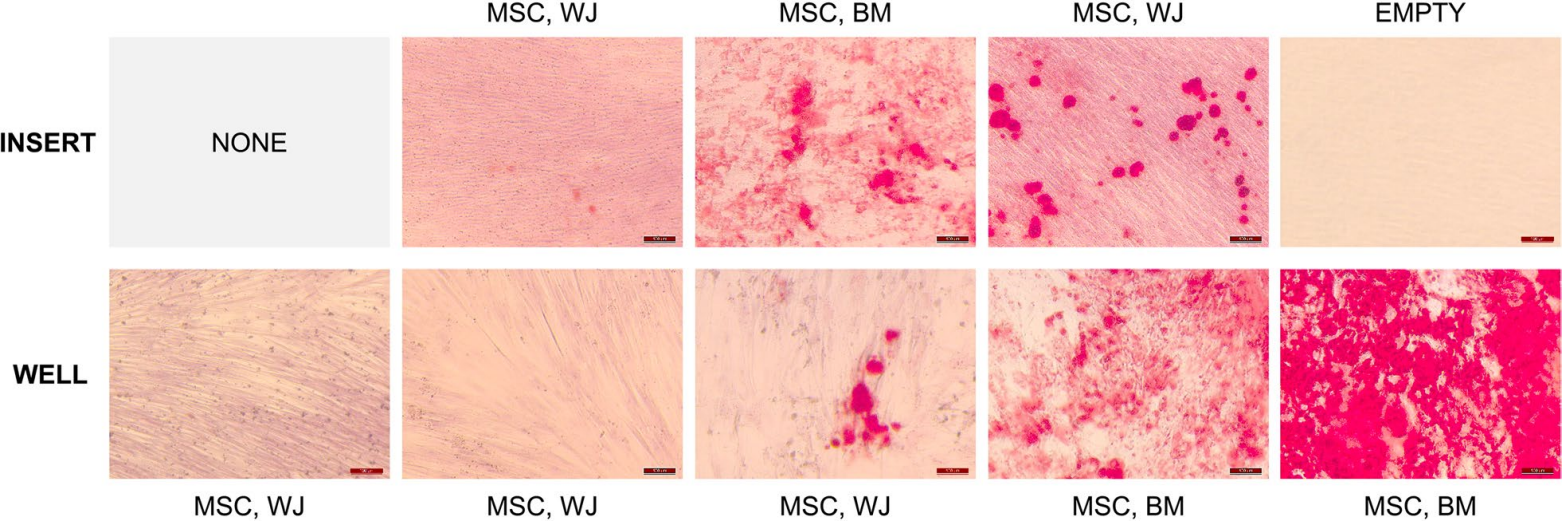
Effect of the MSC, BM secretome on the osteogenic differentiation of MSC, WJ. Representative images of Alizarin Red staining performed on MSC, WJ and MSC, BM cultures after three weeks of in vitro osteogenic induction using adherent transwells. Under these conditions, MSC,WJ and MSC,BM display bone induction, whereas MSC,WJ alone are not capable of committing in the osteogenic lineage in a short time-scale. Scale bar: 100 µm

As shown in Fig. 1, MSC,BM exerted a positive effect on osteogenic differentiation induction of MSC,WJ and resulted in clearly visible calcium deposits by alizarin red (AR) staining three weeks after in vitro culture with osteogenic media. Interestingly, osteogenic differentiation of MSC,WJ was more pronounced when combining MSC,WJ seeded in the insert with MSC,BM seeded on the well surface than when co-cultured in the opposite orientation. In contrast, negative results for AR staining were obtained for the same experimental time point when MSC,WJ were cultured either alone on the well surface or when seeding MSC,WJ in both the insert and the well (Fig. 1). Finally, regarding MSC,BM osteogenic differentiation, calcium depositions in MSC,BM cultures were more abundant when MSC,BM were cultured alone than when they were co-cultured with MSC,WJ (Fig. 1).

### Intratibial injection of MSC,WJ induces new bone formation within the medullary cavity

Our results strongly support that in the presence of the appropriate osteogenic inducers, MSC,WJ can become bone tissue. In an attempt to demonstrate that an intra-bony environment could be enough to allow MSC,WJ-promoted osteogenesis, we directly injected a single dose of 5·10^5^ MSC,WJ (N = 8) or engineered MSC,WJ (eMSC,WJ; N = 8) expressing a luciferase reporter gene (FireFly luciferase [FFly, N = 4] or NanoLuc luciferase [NLuc, N = 4]) into the medullary cavity of tibias of immunodeficient NSG mice. PBS (N = 8), non-treated (NT, N = 3) and MSC,BM (N = 4) groups were also included as controls (see Additional file 1). Six weeks after treatment, the animals were sacrificed, and tibiae were harvested for histological purposes.

**Fig. 2:**
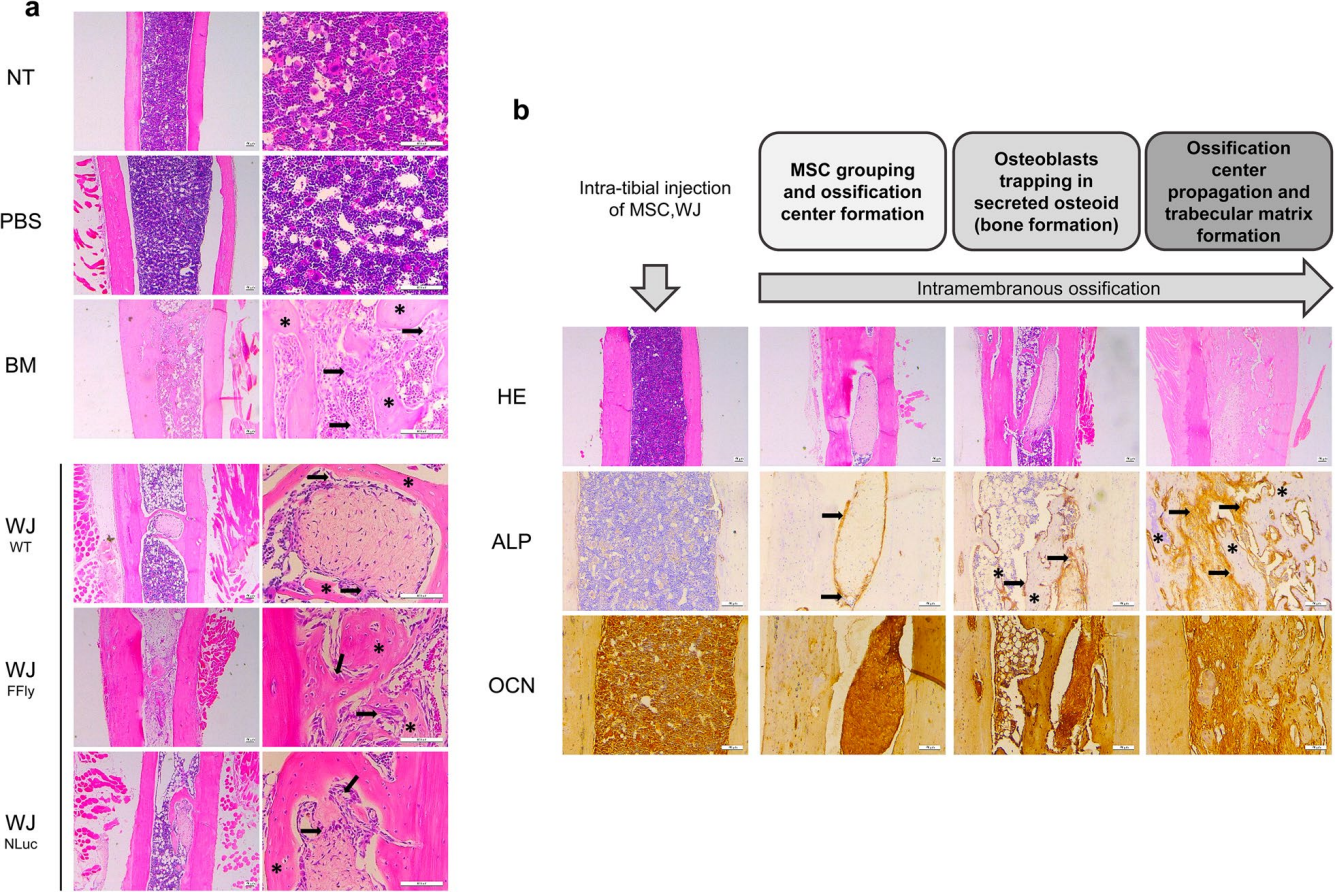
New bone formation within the medullary cavity of NSG mice treated with MSC, WJ. **a** Representative HE staining of longitudinal tibia sections six weeks after treatment. Black arrows indicate the presence of osteoblasts and asterisks indicate new bone trabeculae formed within the medullary cavity. Images in the right column are a magnification of images in the left column. **b** Representative images of HE, ALP, and OCN staining in longitudinal sections of tibias from MSC,WJ-treated animals illustrating the different stages of the intramembranous ossification process promoted by MSC,WJ. Black arrows indicate osteoblasts showing positive expression of ALP and asterisks indicate new bone trabeculae. Scale bar: 100 µm. *NT* non-treated; *WT* wild-type; *FFly* FireFly luciferase; *NLuc* NanoLuciferase; *HE* haematoxylin–eosin; *ALP* Alkaline Phosphatase; *OCN* osteocalcin

Haematoxylin–eosin staining allowed a general view of the structure of the tibia. As can be seen in Fig. 2a, acidic cell components, such as nucleic acids, were positively stained for haematoxylin and appeared purple/dark blue in longitudinal sections. In contrast, basic cellular components and tissues such as cytoplasm, bone, or connective tissue were stained by eosin and appeared in different shades of pink. Signs of bone formation and bone remodelling were observed in the cortical bone for all animals included in the study. This new bone was identified as collagen-rich lacunae (light purple), or disorganized and non-mineralized bone tissue (light pink) embedded in mature cortical bone (dark pink) (Fig. 2a). However, clear differences were observed when comparing the structure and composition of the medullary cavity of animals from different experimental groups.

The medullary cavity of tibias from NT or PBS-treated animals was filled by bone marrow and no other tissue structures were detected in any case (except for the presence of some bone trabeculae originating from the growing cartilage in the proximal area of the tibia, which were present in all experimental groups) (Table 1 and Fig. 2a). In contrast, evidence of new bone formation was clearly seen throughout the medullary cavity of animals treated with MSC,WJ or MSC,BM.

**Table 1:** Bone formation, biodistribution and persistence of MSCs

	*NT*	*PBS*	*MSC,BM*	*MSC,WJ (WT)*	*eMSC,WJ (FFly)*	*eMSC,WJ (NLuc)*
New bone formation detected throughout the medullary cavity						
*6 weeks*	0/3	0/8	1/4	5/8	2/4	3/4
Follow-up of MSC biodistribution and persistence (IVIS bioimaging)						
*48 h*	–	–	–	–	4/4	4/4
*2 weeks*	–	–	–	–	1/4	2/2
*6 weeks*	–	–	–	–	0/4	1/4
MSC biodistribution (amplification of human specific Alu sequences)						
*Brain*	–	–	0/4	0/7	1/4	0/4
*Heart*	–	–	0/4	0/7	0/4	1/4
*Lung*	–	–	2/4	4/7	2/4	3/4
*Liver*	–	–	0/4	0/7	0/4	0/4
*Spleen*	–	–	0/4	0/7	0/4	1/3
*Kidney*	–	–	0/4	0/7	0/4	1/4
*Gonads*	–	–	0/4	0/7	2/4	0/3
Persistence of MSCs in the injection site (human COXIV immunostaining)						
*6 weeks*	–	–	3/4	8/8	2/4	3/4

Summary of results indicating the proportion of animals in each experimental group showing a positive outcome for the specified parameters and experimental time points (indicated as weeks after treatment). NT, non-treated; WT, wild-type; eMSC,WJ, engineered MSC,WJ expressing FireFly luciferase (FFly) or NanoLuc luciferase (NLuc) reporter genes

For MSC,BM-treated mice, intramedullary bone was observed in only one of four mice (25%) (Table 1). In this case, a network of loose connective tissue containing a high density of bone precursor cells and osteoblasts as well as a clear locus of osteoblasts trapped in the secreted osteoid matrix were observed (Fig. 2a). Because of this ossification process, new bone trabeculae were generated between cortical bone occupying a substantial area of the medullary canal.

Conversely, for mice treated with MSC,WJ (either wild-type [WT] or eMSC,WJ expressing FFly or NLuc), evidence of new bone formation within the medullary cavity was detected in ten out of 16 animals (62.5%). This ratio was the same when considering all mice treated with MSC,WJ (10/16) or when considering the MSC,WJ and eMSC,WJ groups separately (5/8 animals in both cases) (Table 1), indicating that genetic engineering of MSC,WJ to induce luciferase reporter gene expression did not disrupt the osteogenic capacity of these cells, as previously confirmed in vitro (see Additional file 2a). Similar to what was observed with MSC,BM, the presence of loose connective tissue containing precursor bone cells and osteoblasts that were trapped in the osteoid matrix promoting new trabecular bone formation was also seen in animals injected with MSC,WJ (Fig. 2a). However, in some of these mice, nodules of dense connective tissue embedding fibroblast-shaped cells and surrounded by new bone trabeculae were also identified throughout the medullary cavity of the tibias (Fig. 2a). As shown in Fig. 2b, cells located in the periphery of these fibrous nodules presented not fibroblast-like but osteoblast morphology and were highly positive for alkaline phosphatase (ALP), a bone biomarker expressed in the early stages of the ossification process. These osteoblastic cells were responsible for synthesizing the osteoid tissue, which consisted of unmineralized fibrous matrix containing osteoblasts that expressed high levels of ALP. As the ossification process progressed, the number of cells expressing ALP increased, and osteoblasts were trapped by the mineralized matrix, becoming osteocytes that formed part of new bone trabeculae. At this stage, ALP expression was maintained in the osteoblast cell line surrounding newly-formed bone but not in the embedded osteocytes (Fig. 2b). Regarding osteocalcin (OCN) expression, a late marker for bone formation, a marked background was observed for the entire bone tissue in all samples. However, the expression of OCN was higher in the fibrous nodules and in the osteoid tissue generated into the medullary cavity of tibias obtained from MSC,WJ-treated mice (Fig. 2b).

### MSC,WJ are safe and persist at the injection site

**Fig. 3:**
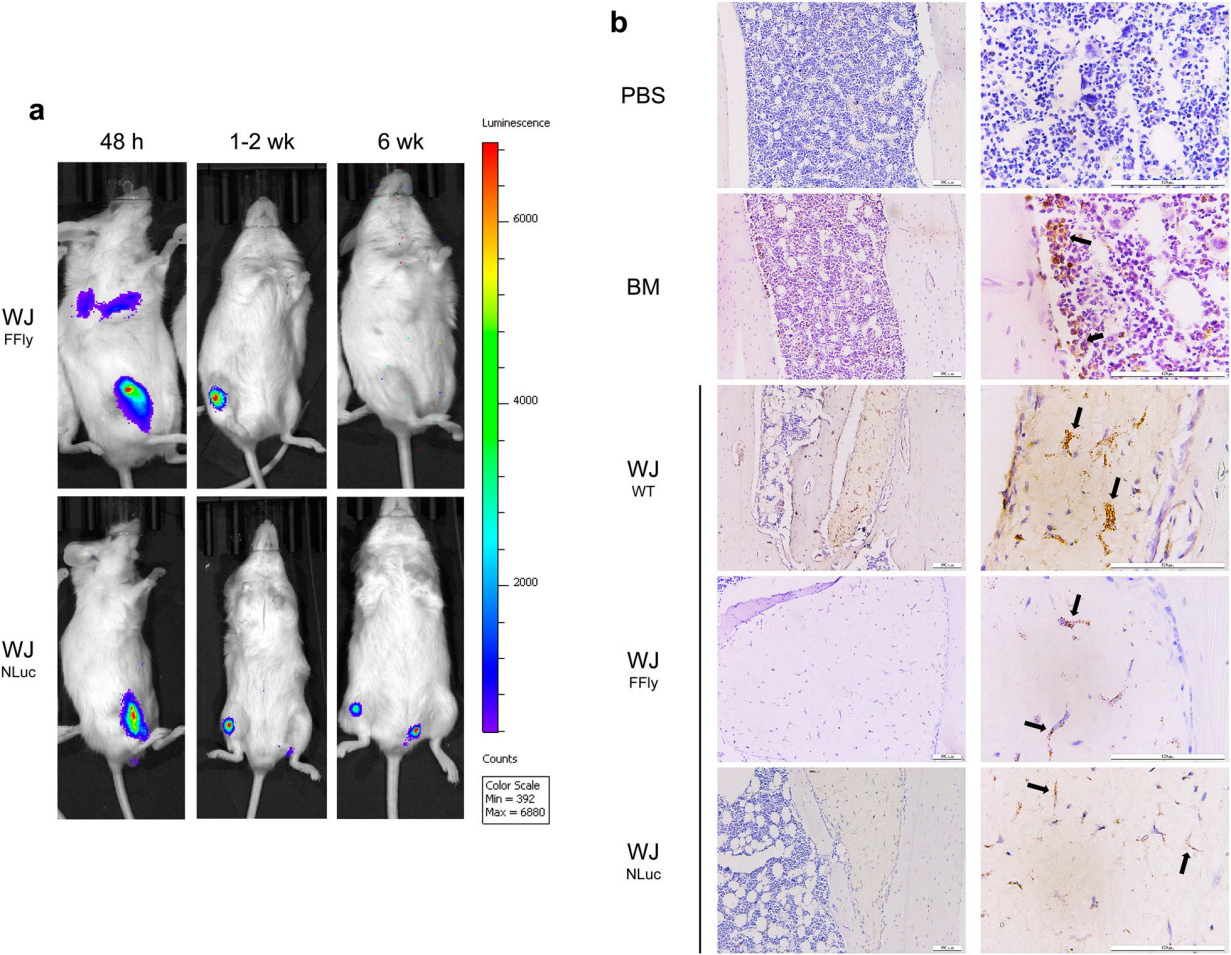
Biodistribution and persistence of xenogeneic MSCs in NSG mice. **a** Representative bioluminescence images showing the biodistribution and persistence of the luminescence produced by FireFly- or NanoLuc-MSC,WJ at 48 h, one-two weeks, and six weeks after treatment. **b** Representative images of human COXIV immunostaining in longitudinal sections of tibias six weeks after treatment. Black arrows indicate positive cells. Images in the right column are a magnification of images in the left column. Scale bar: 100 µm. *WT* wild-type; *FFly* FireFly; *NLuc* NanoLuc

Demonstration of the safety of therapeutic products is essential to translate their use into clinical practice. With this in mind, the biodistribution and persistence of Good Manufacturing Practice (GMP)-grade MSC,WJ was monitored in vivo throughout the experimental period using bioluminescence imaging techniques. In a first attempt, MSC,WJ were genetically modified to obtain eMSC,WJ expressing the FFly luciferase reporter gene (FFly-MSC, WJ) and injected in a second cohort of MSC,WJ-treated NSG mice (N = 4). Forty-eight hours after treatment, a luminescent signal was detected at the injection site for all mice receiving FFly-MSC,WJ, thus confirming that the intratibial administration had been performed correctly (Table 1). At this time, luciferase expression was also observed within the thoracic cavity in one mouse (Fig. 3a). This mouse was the only animal that maintained the FFly luciferase bioluminescence in the right tibia after two weeks. Six weeks after treatment, negative results were also obtained in this individual for the full body (Table 1). In view of these results, a second eMSC,WJ cell line expressing the NLuc luciferase reporter gene was used as a highly sensitive method to monitor cellular biodistribution and persistence in vivo. Forty-eight hours after treatment, all injected mice (N = 4) were positive for NanoLuc luciferase expression in the right tibia, which was maintained for two weeks in the two animals tested (Table 1), thus suggesting that the luminescent signal produced by NanoLuc luciferase would be more stable than that provided by the FireFly. This observation correlated with the results obtained in vitro, which showed higher bioluminescent activity for the NLuc-MSC,WJ cell line (see Additional file 2b). However, the NanoLuc luciferase signal in the injected tibia was only maintained in 1 out of the 4 animals at the end of the study (Table 1 and Fig. 3a), and no signal was detected for any experimental time point in additional body cavities, with the exception of the urinary tract, which was positively marked for some animals independently of the type of luciferase that was injected (FFly or NLuc) or the experimental time point (48 h, two weeks, or six weeks), probably due to the involvement of this system in the metabolism of the luciferase substrates.

Biodistribution and persistence at the injection site of xenogeneic MSCs in NSG mice were also assessed in vitro at the end of the study by quantitative real-time PCR (qPCR) amplification of human-specific Alu sequences and human Cytochrome c oxidase subunit 4 (COXIV) immunostaining, respectively. Regarding biodistribution assays, MSCs of BM and WJ origin were detected in lungs in similar frequencies (2/4 [50%] for MSC, BM and 9/15 [60%] for MSC, WJ). Additionally, cells of human origin were also identified in the brain, heart, spleen, kidney and gonads in some animals treated with eMSC,WJ but not with WT-MSC,WJ or MSC,BM (Table 1). Human COXIV immunostaining in longitudinal sections of treated tibias enabled the detection of human cells in mice injected with MSCs independent of the cell line administered (BM, 3/4; WJ, 8/8; FFly-WJ, 2/4; NLuc-WJ, 3/4) (Table 1 and Fig. 3b). Cells positive for human COXIV were located either dispersed inside the medullary cavity in the bone marrow stroma or within the fibrous nodules surrounded by newly-formed bone trabeculae, which were only observed in MSC,WJ-treated mice (Fig. 3b).

Importantly, no macroscopic histopathological alterations, ectopic bone formation or any other adverse events were detected in MSC-treated mice either during the follow-up period or during the macroscopic necropsy performed at the end of the study, thus demonstrating the safety of administrating human MSC,WJ in immunodeficient mice.

## Discussion

The use of MSCs in clinical trials in regenerative medicine and modulation of the unbalanced immune system has been increasing exponentially since 2005 due to different attributes of this cell type, including their self-renewal capacity that gives them the potential to be readily scaled up for production, their differentiation potential, and their immunomodulatory properties. Remarkably, a considerable number of registered trials focus on treatments in the field of orthopaedics, which includes osteoarthritis, fracture healing, osteonecrosis, and osteoporosis, among others [42]. Interestingly, MSCs display the ability to promote bone regeneration both by cell autonomous osteogenic commitment as well as by paracrine signalling to recruit other cell types (e.g., osteoclasts, endothelial precursors) and modulate the inflammatory phase of tissue regeneration [43].

Osteoporosis is a systemic metabolic bone disease in which the pool of bone cells diminishes with age, and has been recognized as a major public health concern due to its growing incidence as a result of population ageing [44]. MSC transplantation has provided successful evidence of halting the deterioration of the disease in the preclinical setting by enhancing osteogenic differentiation and increasing bone mineral density. Most of the studies are based on the transplantation of allogeneic MSC,BM or ASCs by intra-bone marrow or intravenous administration [45]. However, in the allogeneic context, WJ presents an advantage as a source of MSCs, mainly because their isolation is not associated with painful and invasive procedures that result in donor-site morbidity and because umbilical cord is a newborn tissue and, consequently, MSC,WJ are less immunogenic [23, 46, 47].

The osteogenic differentiation potential of MSC,WJ has been extensively reported in vitro. However, although capable, MSC,WJ are more recalcitrant than MSC from alternative sources to differentiate into the osteogenic lineage in typical osteogenic induction conditions. The results obtained in the transwell experiment demonstrated that the secretome of synchronically induced MSC,BM has a positive effect on the differentiation of MSC,WJ. Interestingly, osteogenic differentiation of MSC,WJ was more pronounced when combining MSC,WJ seeded in the insert with MSC,BM seeded on the well surface than when they were co-coculture in the opposite orientation, probably because in terms of absolute cell numbers, this combination allows for a higher MSC,BM:MSC,WJ ratio and, therefore, a higher concentration of MSC,BM-secreted osteogenic factors in the culture media. Remarkably, differentiation of MSC,BM cultures was greater when MSC,BM were cultured alone than when they were co-cultured with MSC,WJ. This fact would suggests a negative effect of the MSC,WJ’s secretome on the progression of the differentiation process. However, the exacerbated outcome of AR staining in single MSC,BM cultures could also be explained by a higher availability of osteogenic inductors provided by the culture media as when MSC,BM were co-cultured with empty inserts, total media volume (considering well plus insert) and therefore total amount of osteogenic inductors per cell was superior than when cells were cultured in both surfaces. In view of this results, further experiments are intended aim to the identification of potential osteogenic inductors/inhibitors present in MSC,BM and MSC,WJ conditioned media as well as of their mechanism of action.

In the in vivo context, evidence of the efficacy of MSC,WJ to promote bone formation is still limited. In this proof-of-concept study, we demonstrated that direct intratibial injection of xenogeneic MSC,WJ in NSG immunodeficient mice promotes new trabecular bone formation within the medullary cavity. The results obtained are compatible with an intramembranous ossification process in which injected MSCs grouped to form an initial ossification centre; some cells then started to differentiate into osteoblasts that synthesized osteoid tissue and promoted matrix mineralization, being trapped in bone lacunae and becoming osteocytes (bone formation); and finally, the ossification centre propagated, giving rise to a matrix of newly-formed trabecular bone. These findings confirm our hypothesis and support the idea that osteogenic commitment of MSC,WJ is delayed in vitro due to poor replication of the in vivo osteogenic milieu, thus calling into question alternative approaches such as chemical priming of MSC in vitro or genetic engineering to enhance osteogenic lineage commitment for further use in in vivo applications [48–50].

The mechanism of action by which MSCs promote bone regeneration is controversial. Today, following the publication of a considerable number of preclinical studies showing the beneficial effects of using allogeneic MSC haplolines but lacking identification of the administered cells at the treatment site, the most widely accepted hypothesis is that, once homed in the harmed tissue, MSCs activate tissue regeneration by immunomodulatory effects, stimulation of angiogenesis, antiapoptotic effects in osteoblastic lineage cells, recruitment of host MSCs or progenitor cells, and stimulation of their differentiation into osteoblasts, rather than by direct differentiation into tissue functional cells [43]. In this study, human COXIV immunostaining allowed us to detect the infused MSCs in longitudinal sections of the treated tibias. Interestingly, fibrous-shaped cells embedded within the ossification centres observed in MSC,WJ-treated mice were highly positive for human COXIV expression, demonstrating the persistence of the xenogeneic cells at the injection site and their direct implication in the formation of new bone trabeculae. Despite this, expression of human COXIV was not observed in osteoblastic cells, suggesting that host tissue cells promoted by MSC,WJ-secreted factors also take part and play a relevant role in the ossification process. In this case, the persistence and effectiveness of xenogeneic MSCs in immunodeficient mice could be explained by the inability of a deficient immune system characterized by defective innate immunity (absent haemolytic complement system, reduced dendritic cell function, and defective macrophage activity) and lack of mature T cells, B cells, and natural killer cells to detect and eliminate foreign cells. However, previous studies by our group demonstrated that osteogenic stimuli produced by MSC,BM after one week in differentiating culture conditions was enough to induce MSC,WJ osteogenic commitment by paracrine signalling. This suggests that even though un-matched MSCs would be rejected shortly after infusion in immunocompetent patients, they could efficiently induce osteogenic signalling pathways that would enable the recruitment and stimulation of host tissue cells. To overcome the limitation of using an immunodeficient mouse model and confirm this hypothesis, additional studies are currently in progress in an immunocompetent sheep model of cylindrical bone defects treated with allogeneic ovine MSC,WJ.

As already stated, the identification of essential factors accounting for the efficacy of MSC on promoting in vivo osteogenesis is particularly relevant to understand the biological mechanisms involved in the process and must be investigated. In relation to this, our results corroborate our previous findings and demonstrate that the secretome of MSC,BM could be indispensable to ensure a successful outcome when using MSC,WJ in patients with bone-related conditions requiring new bone formation. This could have a marked effect in those cases in which the medical status of the patient might impair or limit osteogenic and immunomodulatory properties of autologous MSCs, as usually happens in elderly osteoporosis patients. In this scenario, the administration of allogeneic MSC,WJ in combination with a set of defined osteogenic inducers might be a more effective therapeutic option.

Intra-bone injection of MSCs was found to be an efficient administration route to achieve new bone formation in NSG mice. However, although local injection of MSCs is the option of choice in clinical practice when they are combined with scaffolds or grafts during an invasive procedure [6, 51], and intraosseous injection of MSCs into the medullary bone cavities has been shown to be feasible and advantageous in indications such as haematopoietic stem cell transplantation to reduce graft-versus-host disease [52, 53], this approach should be further evaluated clinically. Alternatively, peripheral vein infusion of MSCs is a minimally invasive approach, although its use for the treatment of bone diseases could limit the therapeutic benefit if the cells do not reach the affected bones. To overcome this issue, fucosylated (e.g. exofucosylation of CD44 membrane antigen) or genetically modified MSC,WJ (e.g. CXCR4-induced expression) could be used to enhance osteotropism, promote directional homing of MSCs to bone defect sites, and increase bone repair [54, 55]. In this context, a phase I clinical trial for osteoporosis using autologous fucosylated MSC,BM injected intravenously has already been completed (NCT02566655, EudraCT 2012-005814-20) and, even though clinical results are not available, previous preclinical studies in NOD/SCID mice reported that mice receiving intravenous infusions of fucosylated cells showed a higher degree of osteblastogenesis than those infused with non-fucosylated MSC,BM (Cabañas V, Universidad de Murcia, personal communication).

The use of eMSC,WJ expressing luciferase reporter genes allowed us to monitor the systemic biodistribution and persistence of the infused cells in vivo. Regarding the use of FireFly and NanoLuc luciferase, the NanoLuc luciferase luminescent signal seemed to be more stable, as previously reported [56]. However, at the end of the treatment, negative results for bioluminescence imaging were obtained for all except one mouse, despite identifying the persistence of cells in the treated tibias, as shown by COXIV immunostaining. This confirms the limitations of bioluminescence imaging techniques, offering poor tissue penetration and low spatial resolution [57]. Apart from the injected tibias, a transient luminescence signal was observed in the thoracic cavity in one animal. This observation is consistent with the results obtained by amplification of human specific Alu sequences at the end of treatment, which revealed the persistence of human cells in the lung in a large proportion of MSC-treated mice, regardless of the MSC origin. Additionally, qPCR amplification revealed that human MSCs were also present in the brain, heart, spleen, kidney, and gonads of some animals treated with eMSC,WJ. These findings are in agreement with the well-known initial mechanical entrapment of infused MSCs in lung microvasculature and posterior migration to other organs. However, systemic distribution of MSCs is usually described when performing intravenous administration [58]. Thus, although bioluminescence and COXIV immunostaining results indicate that cellular intratibial administration was achieved, a degree of leakage to the vascular system was produced, allowing the migration of MSCs to the specified tissues, which has been already reported especially when injection volumes are over 3 µl [59]. Notably, despite these findings, no adverse events associated with the use of MSCs or macroscopic histopathological alterations were identified in NSG mice. In relation to this, treatment with MSCs from different origins by either local injection or intravenous administration has proven to be safe in clinical trials, and no serious adverse events other than transient fever, administration site adverse events, constipation, fatigue, and sleeplessness have been reported so far [60].

## Conclusions

In conclusion, our study demonstrates the suitability of using MSCs from WJ in the promotion of osteogenesis in vivo without the need to add supplemental osteogenic inducers, and demonstrates that treatment with MSC,WJ can could be a feasible option for patients with bone-related conditions requiring new bone formation.

### Supplementary Information

**Additional file 1: Table S1.** Distribution and justification of the experimental study groups. Composition, nature, and justification of the different experimental treatment groups included in the study. N, number of animals; TI, Test Item; RI, Reference Item; FFly, FireFly; NLuc, NanoLuc; NT, Non-Treated; BLI, Bioluminescence Imaging.

**Additional file 2: Figure S1.** Characterization of engineered MSC,WJ. (a) Representative images of alizarin red staining in eMSC, WJ cultures after five weeks of osteogenic induction in vitro. (b) Bioluminescence activity of eMSC, WJ. eMSC,WJ, engineered MSC,WJ; FFly, FireFLy; NLuc, NanoLuc.

## Data Availability

The data that support the findings of this study are available from the corresponding authors, JV and LB-M, upon reasonable request.

## Abbreviations

ASC: Adipose stem cells
ATMPs: Advanced therapy medicinal products
AR: Alizarin red
ALP: Alkaline phosphatase
BM: Bone marrow
COXIV: Cytochrome c oxidase subunit 4
eMSC,WJ: Engineered MSC,WJ
FFly: Firefly luciferase
GMP: Good manufacturing practice
MSC: Mesenchymal stromal cells
NLuc: NanoLuc luciferase
NT: Non-treated
OCN: Osteocalcin
qRT-PCR: Quantitative real-time PCR
WJ: Wharton’s jelly

## References

[1] Wu AM, Bisignano C, James SL, Abady GG, Abedi A, Abu-Gharbieh E (2021). Global, regional, and national burden of bone fractures in 204 countries and territories, 1990–2019: a systematic analysis from the global burden of disease study 2019. Lancet Heal Longev.

[2] Salari N, Ghasemi H, Mohammadi L, Behzadi MH, Rabieenia E, Shohaimi S (2021). The global prevalence of osteoporosis in the world: a comprehensive systematic review and meta-analysis. J Orthop Surg Res.

[3] Shen Y, Huang X, Wu J, Lin X, Zhou X, Zhu Z (2022). The global burden of osteoporosis, low bone mass, and its related fracture in 204 countries and territories, 1990–2019. Front Endocrinol.

[4] Naji A, Eitoku M, Favier B, Deschaseaux F, Rouas-Freiss N, Suganuma N (2019). Biological functions of mesenchymal stem cells and clinical implications. Cell Mol Life Sci.

[5] Im G (2017). Clinical use of stem cells in orthopaedics. Eur Cells Mater.

[6] Perez JR, Kouroupis D, Li DJ, Best TM, Kaplan L, Correa D (2018). Tissue engineering and cell-based therapies for fractures and bone defects. Front Bioeng Biotechnol.

[7] Fu J, Wang Y, Jiang Y, Du J, Xu J, Liu Y (2021). Systemic therapy of MSCs in bone regeneration: a systematic review and meta-analysis. Stem Cell Res Ther.

[8] Yi H, Wang Y, Liang Q, Mao X (2022). Preclinical and clinical amelioration of bone fractures with mesenchymal stromal cells: a systematic review and meta-analysis. Cell Transplant.

[9] Prat S, Gallardo-Villares S, Vives M, Carreño A, Caminal M, Oliver-Vila I (2018). Clinical translation of a mesenchymal stromal cell-based therapy developed in a large animal model and two case studies of the treatment of atrophic pseudoarthrosis. J Tissue Eng Regen Med.

[10] López-Fernández A, Barro V, Ortiz-Hernández M, Manzanares MC, Vivas D, Vives J (2020). Effect of allogeneic cell-based tissue-engineered treatments in a sheep osteonecrosis model. Tissue Eng - Part A.

[11] de García Frutos A, González-Tartière P, Coll Bonet R, Ubierna Garcés MT, del Arco CA, Rivas García A (2020). Randomized clinical trial: expanded autologous bone marrow mesenchymal cells combined with allogeneic bone tissue, compared with autologous iliac crest graft in lumbar fusion surgery. Spine J.

[12] Gómez-Barrena E, Padilla-Eguiluz NG, Rosset P, Hernigou P, Baldini N, Ciapetti G (2021). Osteonecrosis of the femoral head safely healed with autologous, expanded, bone marrow-derived mesenchymal stromal cells in a multicentric trial with minimum 5 years follow-up. J Clin Med.

[13] Gastelurrutia P, Prat-Vidal C, Vives J, Coll R, Bayes-Genis A, Gálvez-Montón C (2021). Transitioning from preclinical evidence to advanced therapy medicinal product: a spanish experience. Front Cardiovasc Med.

[14] Singh-Mohal J, Tailor HD, Khan WS (2012). Sources of adult mesenchymal stem cells and their applicability for musculoskeletal applications. Curr Stem Cell Res Ther.

[15] Murphy MB, Moncivais K, Caplan AI (2013). Mesenchymal stem cells: environmentally responsive therapeutics for regenerative medicine. Exp Mol Med.

[16] Arthur A, Gronthos S (2020). Clinical application of bone marrow mesenchymal stem/stromal cells to repair skeletal tissue. Int J Mol Sci.

[17] Dimmeler S, Leri A (2008). Aging and disease as modifiers of efficacy of cell therapy. Circ Res.

[18] Baker N, Boyette LB, Tuan RS (2015). Characterization of bone marrow-derived mesenchymal stem cells in aging. Bone.

[19] Karantalis V, Hernandez-Schulman I, Balkan W, Hare JM (2015). Allogeneic cell therapy: a new paradigm in therapeutics. Circ Res.

[20] Grau-vorster M, Laitinen A, Nystedt J, Vives J (2019). HLA-DR expression in clinical-grade bone marrow-derived multipotent mesenchymal stromal cells : a two-site study. Stem Cell Res Ther.

[21] Muñoz-Domínguez N, Carreras-Sánchez I, López-Fernández A, Vives J (2022). Optimisation of processing methods to improve success in the derivation of human multipotent mesenchymal stromal cells from cryopreserved umbilical cord tissue fragments. Cryobiology.

[22] Oliver-Vila I, Coca MI, Grau-Vorster M, Pujals-Fonts N, Caminal M, Casamayor-Genescà A (2016). Evaluation of a cell-banking strategy for the production of clinical grade mesenchymal stromal cells from Wharton’s jelly. Cytotherapy.

[23] Deuse T, Stubbendorf M, Tang-Quan K, Phillips N, Kay MA, Eiermann T (2011). Immunogenicity and immunomodulatory properties of umbilical cord lining mesenchymal stem cells. Cell Transplant.

[24] Liau LL, Ruszymah BHI, Ng MH, Law JX (2020). Characteristics and clinical applications of Wharton’s jelly-derived mesenchymal stromal cells. Curr Res Transl Med.

[25] Davies J, Walker J, Keating A (2017). Concise review : Wharton’s jelly : the rich, but rnigmatic, source of mesenchymal stromal cells. Stem Cells Transl Med.

[26] Abbaszadeh H, Ghorbani F, Derakhshani M, Movassaghpour AA, Yousefi M, Talebi M (2020). Regenerative potential of Wharton’s jelly-derived mesenchymal stem cells: a new horizon of stem cell therapy. J Cell Physiol.

[27] Zajdel A, Kalucka M, Kokoszka-Mikolaj E, Wilczok A (2017). Osteogenic differentiation of human mesenchymal stem cells from adipose tissue and Wharton’s jelly of the umbilical cord. Acta Biochim Pol.

[28] Bharti D, Shivakumar SB, Park JK, Ullah I, Subbarao RB, Park JS (2018). Comparative analysis of human Wharton’s jelly mesenchymal stem cells derived from different parts of the same umbilical cord. Cell Tissue Res.

[29] Ansari AS, Yazid MD, Qisya N, Veronica A, Razali RA, Bin SA (2018). Osteogenic induction of Wharton’s jelly-derived mesenchymal stem cell for bone regeneration : a systematic review. Stem Cells Int.

[30] Hsieh J-Y, Fu Y-S, Chang S-J, Tsuang Y-H, Wang H-W (2010). Mesenchymal stem cells from bone marrow and Wharton’s jelly of umbilical cord. Stem Cells Dev.

[31] Szepesi Á, Matula Z, Szigeti A, Várady G, Szalma J, Szabó G (2016). In Vitro Characterization of human mesenchymal stem cells isolated from different tissues with a potential to promote complex bone regeneration. Stem Cells Int.

[32] Batsali AK, Pontikoglou C, Koutroulakis D, Pavlaki KI, Damianaki A, Mavroudi I (2017). Differential expression of cell cycle and WNT pathway-related genes accounts for differences in the growth and differentiation potential of Wharton’s jelly and bone marrow-derived mesenchymal stem cells. Stem Cell Res Ther.

[33] Midha S, Jain KG, Bhaskar N, Kaur A, Rawat S, Giri S (2021). Tissue-specific mesenchymal stem cell-dependent osteogenesis in highly porous chitosan-based bone analogs. Stem Cells Transl Med.

[34] Chen CF, Chen YC, Fu YS, Tsai SW, Wu PK, Chen CM (2021). Characterization of osteogenesis and chondrogenesis of human decellularized allogeneic bone with mesenchymal stem cells derived from bone marrow, adipose tissue, and wharton’s jelly. Int J Mol Sci.

[35] Kang BJ, Ryu HH, Park SS, Koyama Y, Kikuchi M, Woo HM (2012). Comparing the osteogenic potential of canine mesenchymal stem cells derived from adipose tissues, bone marrow, umbilical cord blood, and Wharton’s jelly for treating bone defects. J Vet Sci.

[36] Hou T, Xu J, Wu X, Xie Z, Luo F, Zhang Z (2009). Umbilical cord Wharton ’ s jelly : a new potential cell source for bobe tissue engineering. Tissue Eng Part A.

[37] Han SM, Han SH, Coh YR, Jang G, Ra JC, Kang SK (2014). Enhanced proliferation and differentiation of Oct4- and Sox2-overexpressing human adipose tissue mesenchymal stem cells. Exp Mol Med.

[38] Saeed H, Qiu W, Li C, Flyvbjerg A, Abdallah BM, Kassem M (2015). Telomerase activity promotes osteoblast differentiation by modulating IGF-signaling pathway. Biogerontology.

[39] Xu L, Huang S, Hou Y, Liu Y, Ni M, Meng F (2015). Sox11-modified mesenchymal stem cells (MSCs) accelerate bone fracture healing: Sox11 regulates differentiation and migration of MSCs. FASEB J.

[40] Cabrera-Pérez R, Monguió-Tortajada M, Gámez-Valero A, Rojas-Márquez R, Borràs FE, Roura S (2019). Osteogenic commitment of Wharton ’ s jelly mesenchymal stromal cells : mechanisms and implications for bioprocess development and clinical application. Stem Cell Res Ther.

[41] Funakoshi K, Bagheri M, Zhou M, Suzuki R, Abe H, Akashi H (2017). Highly sensitive and specific Alu-based quantification of human cells among rodent cells. Sci Rep.

[42] Rodríguez-Fuentes DE, Fernández-Garza LE, Samia-Meza JA, Barrera-Barrera SA, Caplan AI, Barrera-Saldaña HA (2021). Mesenchymal stem cells current clinical applications: a systematic review. Arch Med Res.

[43] Oryan A, Kamali A, Moshirib A, Eslaminejad MB (2017). Role of mesenchymal stem cells in bone regenerative medicine: what is the evidence?. Cells Tissues Organs.

[44] Clynes MA, Harvey NC, Curtis EM, Fuggle NR, Elaine M, Cooper C (2020). The epidemiology of osteoporosis. Br Med Bull.

[45] Jiang Y, Zhang P, Zhang X, Lv L, Zhou Y (2021). Advances in mesenchymal stem cell transplantation for the treatment of osteoporosis. Cell Prolif.

[46] Yoo KH, Jang IK, Lee MW, Kim HE, Yang MS, Eom Y (2009). Comparison of immunomodulatory properties of mesenchymal stem cells derived from adult human tissues. Cell Immunol.

[47] Wang Q, Yang Q, Wang Z, Tong H, Ma L, Zhang Y (2016). Comparative analysis of human mesenchymal stem cells from fetal-bone marrow, adipose tissue, and Warton’s jelly as sources of cell immunomodulatory therapy. Hum Vaccines Immunother.

[48] Rosova I, Nolta JA (2007). Hypoxic preconditioning results in increased motility and improved therapeutic potential of human mesenchymal stem cells in a xenograft hind limb ischemia injury model. Blood.

[49] Bougioukli S, Saitta B, Sugiyama O, Tang AH, Elphingstone J, Evseenko D (2019). Lentiviral gene therapy for bone repair using human umbilical cord blood-derived mesenchymal stem cells. Hum Gene Ther.

[50] Hiew VV, Teoh PL (2022). Collagen modulates the biological characteristics of WJ-MSCs in basal and osteoinduced conditions. Stem Cells Int.

[51] Šponer P, Kučera T, Brtková J, Urban K, Kočí Z, Měřička P (2018). Comparative study on the application of mesenchymal stromal cells combined with tricalcium phosphate scaffold into femoral bone defects. Cell Transplant.

[52] Ramirez PA, Wagner JE, Brunstein CG (2010). Going straight to the point: intra-BM injection of hematopoietic progenitors. Bone Marrow Transplant.

[53] Goto T, Murata M, Nishida T, Terakura S, Kamoshita S, Ishikawa Y (2021). Phase I clinical trial of intra-bone marrow cotransplantation of mesenchymal stem cells in cord blood transplantation. Stem Cells Transl Med.

[54] Sackstein R, Merzaban JS, Cain DW, Dagia NM, Spencer JA, Lin CP (2008). Ex vivo glycan engineering of CD44 programs human multipotent mesenchymal stromal cell trafficking to bone. Nat Med.

[55] Sanghani A, Osagie-Clouard L, Samizadeh S, Coathup MJ, Kalia P, Di Silvio L (2018). CXCR4 has the potential to enhance bone formation in osteopenic rats. Tissue Eng - Part A.

[56] England CG, Ehlerding EB, Cai W (2016). NanoLuc: a small luciferase is brightening up the field of bioluminescence. Bioconjug Chem.

[57] Kim SJ, Lee H-Y (2022). In vivo molecular imaging in preclinical research. Lab Anim Res.

[58] Sanchez-Diaz M, Quiñones-Vico MI, de la Torre RS, Montero-Vílchez T, Sierra-Sánchez A, Molina-Leyva A (2021). Biodistribution of mesenchymal stromal cells after administration in animal models and humans: a systematic review. J Clin Med.

[59] Fink D, Pfeiffenberger U, Bernthaler T, Schober S, Thonhauser KE, Rülicke T (2019). Capacity of the medullary cavity of tibia and femur for intra-bone marrow transplantation in mice. PLoS ONE.

[60] Wang Y, Yi H, Song Y (2021). The safety of MSC therapy over the past 15 years: a meta-analysis. Stem Cell Res Ther.

