# Supplementary material for: Human Wharton’s jelly-derived mesenchymal stromal cells promote bone formation in immunodeficient mice when administered into a bone microenvironment
Source: J Transl Med. 2023 Nov 10;21:802. doi: 10.1186/s12967-023-04672-9 (PMC10638709; doi:10.1186/s12967-023-04672-9)
Supplement: Supplementary file 1 — Additional file 1: Table S1. Distribution and justification of the experimental study groups. Composition, nature, and justification of the different experimental treatment groups included in the study. N, number of animals; TI, Test Item; RI, Reference Item; FFly, FireFly; NLuc, NanoLuc; NT, Non-Treated; BLI, Bioluminescence Imaging. [file 12967_2023_4672_MOESM1_ESM.docx]

***ADDITIONAL FILES***

***Additional file 1. Distribution and justification of the experimental study groups.*** Composition, nature, and justification of the different experimental treatment groups included in the study. N, number of animals; TI, Test Item; RI, Reference Item; FFly, FireFly; NLuc, NanoLuc; NT, Non-Treated; BLI, Bioluminescence Imaging.

| **Treatment** | **N** | **Item type** | **Aim** |
| --- | --- | --- | --- |
| MSC,WJ | 8 | TI | To evaluate osteogenesis promoted by MSC,WJ; study of the persistence and biodistribution of MSC,WJ at endpoint |
| FFly-MSC,WJ | 4 | TI | Study of the persistence and biodistribution of MSC,WJ *in vivo* by BLI |
| NLuc-MSC,WJ | 4 | TI | To enhance the bioluminescent signal provided by FFly luciferase |
| PBS | 8 | RI | To evaluate osteogenesis promoted by intratibial injection |
| MSC,BM | 4 | RI | To evaluate osteogenesis promoted by MSCs from BM, which are defined as permissive MSCs for bone differentiation |
| NT | 3 | RI | To evaluate wild-type structure and osteogenesis in non-injected tibia |
