# Supplementary figures and images for: Human Wharton’s jelly-derived mesenchymal stromal cells promote bone formation in immunodeficient mice when administered into a bone microenvironment
Source: J Transl Med. 2023 Nov 10;21:802. doi: 10.1186/s12967-023-04672-9 (PMC10638709; doi:10.1186/s12967-023-04672-9)

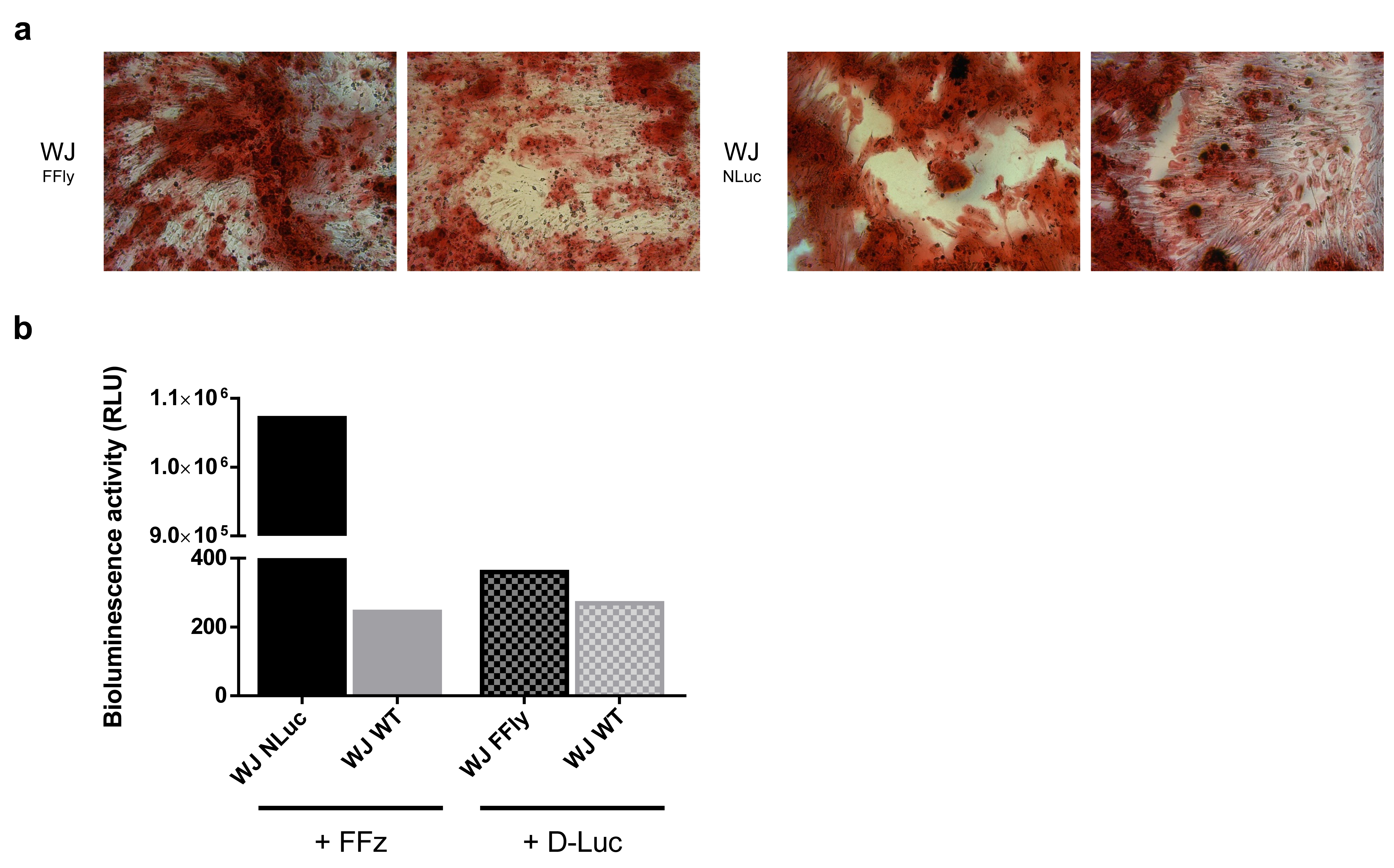

Supplement: Supplementary file 2 — Additional file 2: Figure S1. Characterization of engineered MSC,WJ. (a) Representative images of alizarin red staining in eMSC, WJ cultures after five weeks of osteogenic induction in vitro. (b) Bioluminescence activity of eMSC, WJ. eMSC,WJ, engineered MSC,WJ; FFly, FireFLy; NLuc, NanoLuc. [file 12967_2023_4672_MOESM2_ESM.jpg]
